# Glyphosate affects persistence and tolerance but not antibiotic resistance

**DOI:** 10.1186/s12866-023-02804-1

**Published:** 2023-03-07

**Authors:** Katia Ospino, Beny Spira

**Affiliations:** Departamento de Microbiologia, Instituto de Ciências Biomédicas, Universidade de São Paulo, São Paulo, SP Brazil

**Keywords:** Glyphosate, Antibiotics, Escherichia coli, (p)ppGpp

## Abstract

Glyphosate is a herbicide widely used in food production that blocks the synthesis of aromatic amino acids in plants and in microorganisms and also induces the accumulation of the alarmone (p)ppGpp. The purpose of this study was to investigate whether glyphosate affects the resistance, tolerance or persistence of bacteria towards three different classes of antibiotics and the possible role of (p)ppGpp in this activity. Glyphosate did not affect the minimum inhibitory concentration of the tested antibiotics, but enhanced bacterial tolerance and/or persistence towards them. The upshift in ciprofloxacin and kanamycin tolerance was partially dependent on the presence of *relA* that promotes (p)ppGpp accumulation in response to glyphosate. Conversely, the strong increase in ampicillin tolerance caused by glyphosate was independent of *relA*. We conclude that by inducing aromatic amino acid starvation glyphosate contributes to the temporary increase in *E. coli* tolerance or persistence, but does not affect antibiotic resistance.

**Supplementary Information:**

The online version contains supplementary material available at 10.1186/s12866-023-02804-1.

## Introduction

Bacterial susceptibility to known antibiotics has been steadily dwindling. In addition to intrinsic and acquired resistance through several different molecular mechanisms, such as target modification, efflux pump, enzyme inactivation, and membrane impermeability [1], non-resistant bacteria can temporarily evade the action of antibiotics through physiological processes known as tolerance and persistence. Unlike resistance, tolerance and persistence are transient phenomena in which the level of antibiotic lethality depends on the length of exposure and not on the concentration of the antimicrobial drug [2]. To achieve the death of tolerant and persistent bacteria the duration of antibiotic treatment must be longer than usually required to kill otherwise sensitive bacteria [3]. The biological processes that govern the acquisition of tolerance and/or persistence are influenced by different factors such as genetic background, nutrient availability and other environmental conditions [4]. For instance, mutations in *hipA*, *hipB* and *metG* trigger the stringent response, which inhibits bacterial growth and results in increased tolerance to $\beta$-lactams and fluoroquinolones [5–8].

While tolerance is defined as a status through which the entire population is capable of circumventing immediate killing by antibiotics, “persistence” is a condition in which only a small fraction of the population retains this ability [9]. Therefore, populations bearing persistent bacteria are heterogeneous [10] and present a bimodal time-kill curve [11].

Bacteria are frequently exposed to a plethora of environmental stresses that end up triggering the stringent response, which is mediated by the alarmones guanosine tetraphosphate (ppGpp) and guanosine pentaphosphate (pppGpp) collectively known as (p)ppGpp [12]. (p)ppGpp accumulates in the cell in response to amino acid, carbon, iron, nitrogen, or phosphate shortage, causing a drastic reduction in cell growth [13–18]. However, (p)ppGpp accumulates to higher levels under amino acid starvation than under any other nutritional stress. Under this condition, bacterial growth is completely arrested in order to avoid amino acid imbalance that might result in the production of defective proteins and cell death [19].

(p)ppGpp is associated with tolerance and persistence towards $\beta$-lactams and fluoroquinolones [6–8, 20–25]. If the cell is at a non-growing state due to high levels of (p)ppGpp there are less active molecular targets available for interacting with the antibiotics and thus the bacteria become tolerant.

Glyphosate (N-phosphonomethyl glycine) is one of the most widely used herbicides in the world [26]. It is a weak organic acid that inhibits the synthesis of aromatic amino acids, by inhibiting the enzyme 5-enolpyruvylshikimic acid- 3-phosphate synthase (EPSPS) [27]. This enzyme catalyses the production of chorismate, required for the biosynthesis of phenylalanine, tyrosine, and tryptophan in the biosynthetic shikimate pathway, present only in plants and microorganisms [28, 29]. By leaching, run-off, or overspray, residual levels of glyphosate and its degradation products may reach aquatic and terrestrial ecosystems potentially affecting these communities [30]. Several studies have explored the impact of glyphosate on different microbial communities, sometimes with conflicting results [31–35]. These differences can be attributed to several factors, among which are the type of formulation or dose used, time of exposure, environmental conditions of the microbiome (amino acid availability for instance), and microbial composition [31, 32]. The effect of glyphosate on microbial susceptibility to antibiotics has also been reported [36–38]. Kurenbach *et al.* showed that exposure to Round-up, a commercial formulation of glyphosate, elevated the minimal inhibitory concentration (MIC) of some antibiotics,  such as kanamycin and ciprofloxacin (but not of ampicillin, chloramphenicol or tetracycline) in both *E.coli* and *Salmonella typhimurium* [36], while Costa *et al.* described an evolution experiment in which bacterial communities exposed for long periods of time to relatively high glyphosate concentrations are selected for the presence multidrug efflux pumps that are presumably effective against glyphosate and antibiotics [37]. In addition, it has been shown that exposure to glyphosate increases the prevalence of antibiotic resistance genes and mobile genetic elements by enriching the presence of these elements in the soil microbiome [39]. Altogether, these studies suggest that glyphosate may indirectly promote the dissemination of antibiotic resistance. Experiments performed in our laboratory showed that glyphosate stimulates the accumulation of (p)ppGpp in *E.coli* in a *relA*-dependent fashion, as expected for bacteria starved for amino acids [40]. (p)ppGpp accumulation occurs because glyphosate inhibits the synthesis of aromatic amino acids [27] which in turn induces the synthesis and accumulation of (p)ppGpp [41].

In light of the controversy over glyphosate potential harms and its importance to modern agriculture, this study explored whether glyphosate influences antibiotic resistance, tolerance or persistence towards a $\upbeta$-lactam (ampicillin), a quinolone (ciprofloxacin) and an aminoglycoside (kanamycin). In addition we examined whether (p)ppGpp plays a role in these phenomena.

## Methods

### Bacterial strains

MG1655 was the wild-type *E. coli* K-12 prototype and its derivatives KO1 (MG1655 $\Delta {relA}$) was obtained in this study. Strain MG1655 $\Delta {relA}$::*cat* was constructed by $\uplambda$-red recombination using strain BW25113 carrying plasmid pKD46 [42]. The $\Delta {relA}$::*cat* marker was transferred to strain MG1655 by P1 transduction as described [43]. The *cat* cassette was eliminated through the use of the flippase gene from plasmid pCP20 [42], resulting in strain K01.

### Growth conditions and chemicals

The culture media used in this study were Lysogeny broth (LB)/ Lysogeny Agar (L-agar) [43], and the minimal media TGP [44] and MOPS [45]. Bacteria stored at -80$^{\circ }$C were streaked on L-agar and incubated overnight at 37$^{\circ }$C. The plates were kept at 4$^{\circ }$C until use, but no longer than one week. Glyphosate technical grade (94.5%) was a gift by Monsanto, Brazil. The solution was kept at a stock concentration of 80 mM and stored at room temperature. The antibiotics used in this study were the fluoroquinolone ciprofloxacin (Inlab), the aminoglycoside kanamycin (Inlab), and the $\upbeta$-lactam ampicillin (Inlab). Stock solutions were stored at -20$^{\circ }$C  except for ampicillin that was prepared freshly. All of these compounds were dissolved in milli-Q water and filtered through a 0.2 $\upmu$m filter. In experiments that involved the use of ampicillin, bacteria were grown in MOPS minimal medium. In all other cases, TGP was used.

### Cell viability assay

The effect of glyphosate on bacterial viability was tested as follows: bacteria were grown overnight in LB medium. On the next day, they were washed twice in saline and the optical density of the culture (DO_600_) was adjusted to 3.0 – approximately $3\times 10^9$ bacteria/ml. Then, the bacteria were diluted to a final concentration of $3\cdot 10^3$ cells/ml in one of the following solutions: 10 ml saline (control), 10 ml saline containing 5 mM glyphosate, or 10 ml saline containing 30 mM Tris and 5 mM glyphosate. The bacterial suspensions were incubated at 37$^{\circ }$C under agitation (180 rpm), samples were taken at 0, 5, 15, 30, 60, 120, 180, and 240 minutes and spread on L-agar. The plates were incubated overnight at 37$^{\circ }$C. On the next day, the number of CFU was counted and the proportion of bacterial survival was calculated. The assay was performed with 3 biological replicates.

#### Minimum inhibitory concentration

The MIC was established for the antibiotics ciprofloxacin, kanamycin, and ampicillin and for glyphosate. Overnight cultures were diluted 100-fold in TGP or MOPS minimal medium to an approximate OD_600_ of 0.6 ($\sim 6\cdot 10^8$ bacteria/ml). Bacteria were then diluted 6-fold and 10 $\upmu$L of this suspension were inoculated onto 96-well plates containing 90 $\upmu$l of minimal medium supplemented with one of the antibiotics or glyphosate to obtain a final concentration of $10^7$ bacteria/ml. The plates were incubated at 37$^{\circ }$C for 16-18 hours under agitation (180 rpm). The MIC was estimated by measuring the absorbance at 600 nm using an EPOCH^TM^ spectrophotometer. The MIC was defined as the minimum concentration of antibiotic needed to inhibit 90% of bacterial growth. Each experiment was performed with 3 biological replicates.

#### Chequerboard assay

To determine the simultaneous effect of two compounds on bacterial growth a chequerboard assay was conducted [46]. Serial dilutions of antibiotics and glyphosate were applied to a 96-well plate as shown in Fig. S1. Wells were filled with increasing glyphosate dilutions from columns 1 to 11 and increasing antibiotic dilutions from rows A to G. The following concentrations were used for ampicillin: 32, 16, 8, 4, 2, 1, 0.5, 0.25 $\upmu$g/ml; ciprofloxacin: 0,32, 0.16, 0.08, 0.04, 0.02, 0.01, 0.005, 0.002 $\upmu$g/ml; kanamycin: 16, 8, 4, 2, 1, 0.5, 0.25, 0.12 $\upmu$g/ml and glyphosate: 1690, 845, 422, 221, 111, 56 $\upmu$g/ml. Column 12 and row H were reserved to measure the MIC of each compound alone. The bacterial inoculum and plate reading were done as described above for the determination of the MIC. The results were calculated using the Fractional Inhibitory Concentration ($FIC_{index}$) [47, 48], as follows:

$$\begin{aligned} FIC_{index}= FIC_{A}+FIC_{B}\qquad FIC_{A}=\frac{MIC_{A+Gly}}{MIC_{A}}\qquad FIC_{B}=\frac{MIC_{B+Gly}}{MIC_{B}} \end{aligned}$$

where $MIC_{A+Gly}$ and $MIC_{B+Gly}$ are the MIC of each antibiotic in combination with glyphosate, while $MIC_A$ and $MIC_B$ correspond to the MIC of each compound individually. The chequerboard was performed with 3 biological replicates.

#### Time-kill curves

Bacteria were grown overnight and diluted 100-fold in 2 ml of minimal medium. When cultures attained an OD_600_ of 0.5 they were diluted 50-fold in 12-well plates containing one of the following: culture medium TGP or MOPS (positive control); culture medium containing a 20$\times$ MIC concentration of one of the following antibiotics: kanamycin, ciprofloxacin, or ampicillin; culture medium containing 5 mM glyphosate and one of the antibiotics at a $20\times$ MIC concentration. Bacteria were pre-exposed for 30 minutes to 5 mM glyphosate before adding the antibiotic. The experiment was carried out in a rotator shaker at 200 rpm and 37$^{\circ }$C. Samples were taken at different time intervals of time depending on the antibiotic tested and seeded directly on L-agar plates or diluted in 0.9% NaCl and then plated. CFU were counted on the next day. The assay was performed with at least 3 biological replicates.

### Statistical analysis

Time-kill curves were analysed using R. Two-way repeated-measures ANOVA were performed to evaluate statistically significant differences between treatments in each strain, pairwise t-test was done as a *post-hoc* test. The MDK_99_ and MDK_99.99_ were determined from the time-kill curves using the R *drc* package. This package performs the fitting of non-linear regression models for dose-response analysis.

## Results

### MIC determination of glyphosate, ciprofloxacin, kanamycin, and ampicillin

The principal aim of this study was to clarify whether glyphosate is involved in antibiotic resistance and if (p)ppGpp plays a role in this process. As a first step, the minimal inhibitory concentrations of three antibiotics – ampicillin, ciprofloxacin and kanamycin, each representing a different class of antibiotics, and of glyphosate were evaluated. Because glyphosate affects bacteria by inhibiting the synthesis of aromatic amino acids the MIC assays were performed under non-standard conditions, namely in minimal medium. The MICs were assessed in the wild-type strain MG1655 and in its isogenic $\Delta {relA}$ mutant. For glyphosate, ciprofloxacin and kanamycin, assays were performed in TGP medium, while for ampicillin, MOPS medium was used. Although TGP is the standard medium in our laboratory, ampicillin had to be tested in MOPS medium because ampicillin’s MIC in bacteria grown in TGP was exceptionally high and displayed great variability (data not shown). This unexpected result may be related to the presence of Tris in medium TGP, which forms a complex with Zinc that catalyses penicillin degradation [49]. The two other antibiotics - ciprofloxacin and kanamycin, and glyphosate were not affected by Tris. The MICs of the antibiotics and of glyphosate are summarized in Table 1.

**Table 1:** MIC of ampicillin, ciprofloxacin, kanamycin and glyphosate in the wild-type and in its isogenic $\Delta {relA}$ mutant

	MIC ($\upmu$g/mL)	
Compound	MG1655	$\Delta {relA}$
Ampicillin	2-4	2-4
Ciprofloxacin	0.04	0.04
Kanamycin	1-2	1-2
Glyphosate	845^1^	845

^1^Equivalent to 5 mM

### Interaction of ciprofloxacin, kanamycin, and ampicillin with glyphosate and its effect on antibiotic resistance

The next step was to test whether addition of glyphosate to a bacterial culture would alter the MIC of the tested antibiotics. In order to accomplish this aim, a chequerboard assay with ciprofloxacin, kanamycin, and ampicillin, each one in combination with different concentration of glyphosate, was conducted. The results were calculated using the Fractional Inhibitory Concentration ($FIC_{index}$) [47, 48], which determines whether the interaction between glyphosate and the antibiotics is synergistic ($FIC \le 0.5$), antagonistic ($FIC \ge 4$), or indifferent ($FIC > 0.5\ \textrm{and}\ < 4$). Table 2 shows an **indifferent** interaction of glyphosate with each of the tested antibiotics (ampicillin, ciprofloxacin, or kanamycin) ($FIC_{index} = 2$). These results indicate that glyphosate does not affect the MIC of these antibiotics in *E. coli*.

**Table 2:** Effect of glyphosate on the MIC of *E. coli* toward ampicillin, ciprofloxacin and kanamycin

Combinations	$FIC_{index}$	Interaction
Ampicillin + Glyphosate	2	Indifferent
Ciprofloxacin + Glyphosate	2	Indifferent
Kanamycin + Glyphosate	2	Indifferent

### The effect of glyphosate on the MDK of bacteria treated with antibiotics

**Fig. 1:**
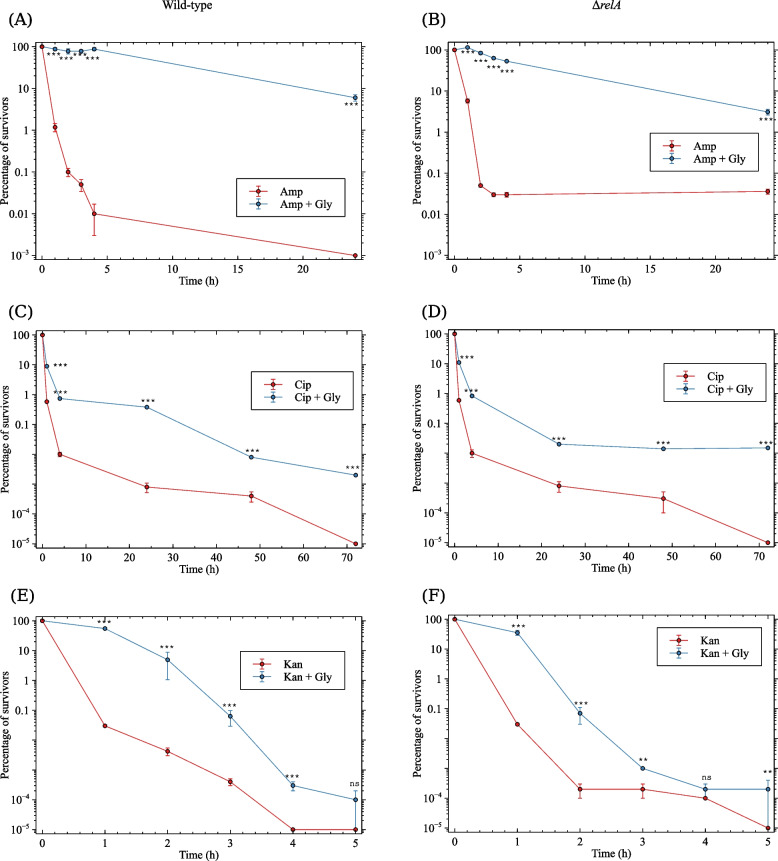
Effect of glyphosate on the MDK of bacteria treated with ampicillin, ciprofloxacin or kanamycin. Exponentially growing cells (OD_600_$\sim$ = 0.5) were diluted to approximately $10^7$ bacteria/ml. 5 mM glyphosate were added to one-half of the cultures and 30 minutes later a final concentration of $20 \times$ MIC of each antibiotic was added to the cultures. Samples were withdrawn at time 0 and at different time-intervals, depending on the antibiotic treatment. Each point corresponds to the mean of at least three independent experiments ± standard deviation (SD). The fraction of survivors was determined by colony counting on L-agar plates. Left (**A**, **C** and **E**), MG1655; Right (**B**, **D** and **F**), MG1655 $\Delta {relA}$. *p*-values were calculated using a pairwise *t*-test between the antibiotic and antibiotic+glyphosate treatment at each time interval. *** indicates $p \le 0.001$; ** indicates $p \le 0.01$; *ns*, not significant

Once the results of the chequerboard showed no effect of glyphosate on bacterial resistance to antibiotics, we proceeded to determine whether the combination of antibiotics with glyphosate could alter the pattern of tolerance and/or persistence. A suitable method for gauging tolerance is the time-kill curve assay [3] and the minimum duration for killing (MDK), a standard metric for measuring tolerance [2, 11]. This method assesses the time it takes to kill a significant proportion of a bacterial population (90%, 99%, 99.99%, and so on) in response to a high dose of an antibiotic. The MDK was obtained by calculating the regression of a time-kill curve [50]. Time-kill curves for ampicillin, ciprofloxacin and kanamycin in the presence of 5 mM glyphosate were conducted (Fig. 1 A, C and E). Antibiotic concentration was in each case $20\times$ the MIC. It can be seen that glyphosate significantly increased cell survival throughout the course of the treatment.

The MDK_99_ and the MDK_99.99_ (minimum duration of time needed to kill 99% and 99.99% of the population, respectively) were calculated from the data obtained in the time-kill curves. The MDKs of the antibiotic+glyphosate treatments were compared to that of the antibiotic alone. Values of MDK_99_ and MDK_99.99_ that are higher in the presence of glyphosate indicate a positive effect of the herbicide on bacterial tolerance. In contrast, the presence of persisters in the population can be attested by higher MDK_99.99_ but similar MDK_99_ values. Addition of glyphosate to ampicillin-treated bacteria strongly affected cell viability, resulting in a significantly milder downward survival curve (Fig. 1A). The MDK_99_ and MDK_99.99_ respectively increased 30 times and 14.6 times (Table 3), suggesting that glyphosate strongly protected the bacteria from the action of ampicillin by increasing their tolerance to the antibiotic.

Unlike the pattern observed for ampicillin, the killing rate of ciprofloxacin-treated cells generated a bimodal curve, which is typical of bacterial cultures that carry persisters. Figure 1C shows that glyphosate reduced cell death at all measured time intervals following the onset of ciprofloxacin treatment. Addition of glyphosate significantly increased tolerance (as evidenced by the slower killing rate in the first 5 hours and a 4.15$\times$ increase in survival) (Table 3), but even more so persistence (bimodal and slower cell death from 5 hours onward with an 11.4$\times$ increase in survival in the presence of glyphosate). Finally, glyphosate slightly suppressed the death of kanamycin-treated bacteria increasing thus the tolerance towards this antibiotic. The time-kill curve of kanamycin-treated cells displayed a bimodal shape, but the biphasic behaviour was only achieved after a 4-log reduction in viability. Since persistence is defined as the time required to achieve a 4-log reduction in viability [11], our data do not support the presence of persisters in the culture. Therefore, we considered that glyphosate conferred a mild increase in tolerance towards kanamycin.

### Glyphosate effect on tolerance and persistence is only partially dependent on (p)ppGpp accumulation

Glyphosate induces the stringent response [40], which is characterised by cell growth arrest, a necessary step in the development of tolerance or persistence [51]. In addition, (p)ppGpp has been directly implicated with both phenomena [6–8, 20–25]. Therefore it is possible that the strong effect of glyphosate on tolerance and persistence towards the three tested antibiotics is associated with the accumulation of (p)ppGpp. We thus asked whether (p)ppGpp might be involved in the process of glyphosate-induced tolerance or persistence against ampicillin, ciprofloxacin and kanamycin. To test this possibility, time-kill curves were performed in the $\Delta {relA}$ mutant, that does not accumulate (p)ppGpp in response to glyphosate [40].

The killing curve of ampicillin-treated $\Delta {relA}$ cells (Fig. 1B) was similar to that of the wild-type strain, a unimodal curve pattern which resulted in a MDK_99_ and MDK_99.99_ of 1.40 and 4.24 h, respectively (Table 3). Unexpectedly, the glyphosate protective effect was even stronger in the $\Delta {relA}$ mutant compared to the wild-type strain – the MDK_99_ and MDK_99.99_ were, respectively, twice and eight times as high as those observed for the wild-type strain (75.1 h vs. 31.46 h and 1789 h vs. 59.7 h). Additionally, the strong increase in the MDK_99.99_ (of 422$\times$) indicates the formation of persister cells in the $\Delta {relA}$ genetic background. This indicate that (1) the strong increase in tolerance in glyphosate-treated *E. coli* does not depend on *relA* and that, contrary to the expectation, (2) glyphosate-induced (p)ppGpp accumulation partially undermines the protective effect of glyphosate. In any case, it is clear that both the wild-type strain and the $\Delta {relA}$ mutant greatly benefited from the growth-inhibiting effect of glyphosate [40].

Glyphosate increased the viability of ciprofloxacin-treated $\Delta {relA}$ bacteria similarly to what has been observed for the wild-type strain – a 4-fold increase in MDK_99_. On the other hand, while the MDK_99.99_ increased by 6.7 times in the $\Delta {relA}$ mutant, in the wild-type strain the effect of glyphosate was slightly higher – 11.4 times. As with the wild-type strain, glyphosate antagonized ciprofloxacin killing by increasing both tolerance and persistence. The fact that the level of persistence (MDK_99.99_) conferred by glyphosate was slightly higher in the wild-type strain than in the $\Delta {relA}$ mutant suggests that the effect of glyphosate is partially dependent on *relA*. Glyphosate increased the MDK_99_ and the MDK_99.99_ of the kanamycin-treated $\Delta {relA}$ strain by 2.6 and 2.1 times, respectively. Similarly to what was observed in ciprofloxacin-treated bacteria, the protective effect of glyphosate against kanamycin was higher in the wild-type strain than in the $\Delta {relA}$ mutant (by 60% and 38% ), suggesting that the increase in tolerance elicited by glyphosate is also partially dependent on *relA*.

**Table 3:** MDK_99_ and MDK_99.99_ of strains MG1655 and $\Delta {relA}$ treated with ampicillin, ciprofloxacin or kanamycin in the presence of glyphosate (Gly). The MDK was measured in hours. Data was calculated from non-linear regression curves derived from the plots shown in Fig. 1

	MG1655			$\Delta {relA}$		
	**Amp**	**Amp+Gly**	$\frac{\mathrm {Amp+Gly}}{\textrm{Amp}}$	**Amp**	**Amp+Gly**	$\frac{\mathrm {Amp+Gly}}{\textrm{Amp}}$
MDK_99_	1.05	31.46	30	1.40	75.1	53.6
MDK_99.99_	4.07	59.70	14.6	4.24	1789	422
	**Cip**	**Cip+Gly**	$\frac{\mathrm {Cip+Gly}}{\textrm{Cip}}$	**Cip**	**Cip+Gly**	$\frac{\mathrm {Cip+Gly}}{\textrm{Cip}}$
MDK_99_	0.83	3.46	4.15	0.83	3.20	3.9
MDK_99.99_	3.76	42.83	11.4	4.29	28.77	6.7
	**Kan**	**Kan+Gl**y	$\frac{\mathrm {Kan+Gly}}{\textrm{Kan}}$	**Kan**	**Kan+Gl**y	$\frac{\mathrm {Kan+Gly}}{\textrm{Kan}}$
MDK_99_	0.57	2.40	4.2	0.56	1.48	2.6
MDK_99.99_	1.13	3.23	2.9	1.13	2.33	2.1

### Glyphosate is bacteriostatic in both *relA*^+^ and $\Delta {relA}$ strains

**Fig. 2:**
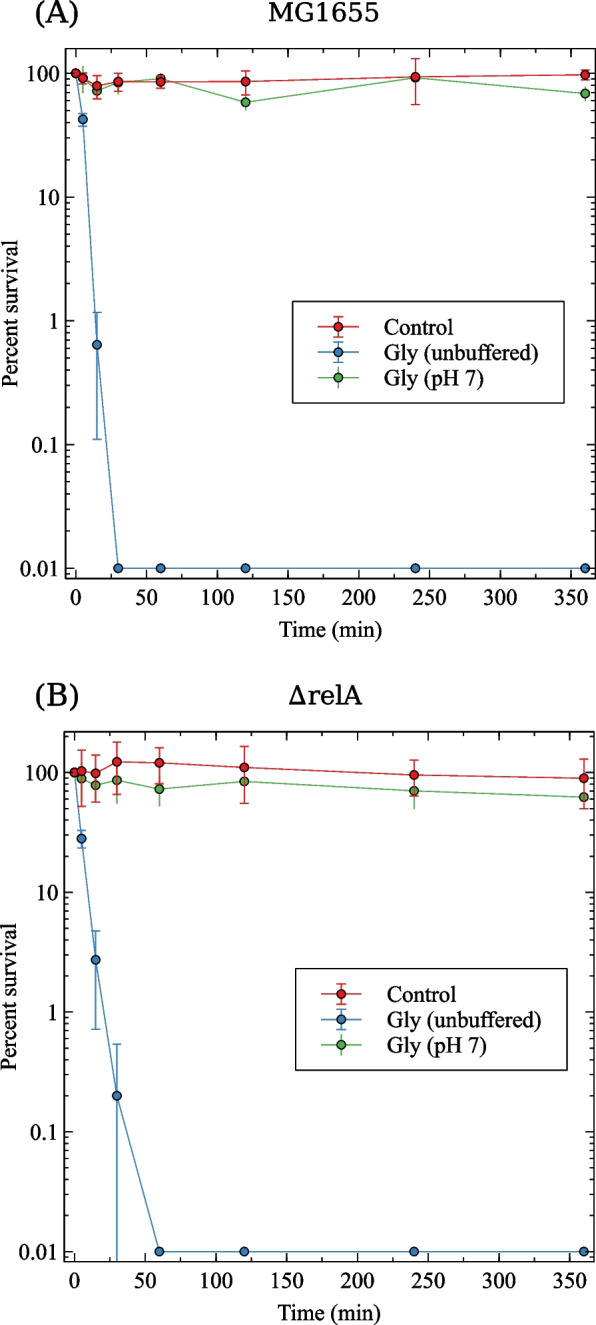
Glyphosate has a bacteriostatic effect even in the absence of *relA*. Exponentially growing $3 \times 10^3$ cells/ml were exposed to 5 mM glyphosate suspended in 0.9% NaCl or in 0.9% NaCl containing 30 mM Tris (pH 7) up to 6 hours at 37$^{\circ }$C. Bacteria suspended in 0.9% NaCl were used as a control. The fraction of survivors was determined by L-agar plating and colony counting. **A** MG1655; **B** MG1655 $\Delta {relA}$. Each point corresponds to the mean of three independent cultures ± standard deviation (SD)

We have previously shown that glyphosate displays a bacteriostatic effect on *E. coli* and that this effect could be reversed by adding aromatic amino acids to the growth medium [40]. However, some antibacterial compounds are bacteriostatic in a (p)ppGpp^+^ strain, but bactericidal in mutants that do not accumulate this alarmone. For instance, chloramphenicol and tetracycline that are considered bacteriostatic antibiotics become bactericidal in *B. subtilis* and *E. faecalis* mutants unable to produce (p)ppGpp [52]. We therefore asked whether glyphosate behaves as a bactericide in the $\Delta {relA}$ mutant, that does not accumulate (p)ppGpp [40]. To test this possibility, the wild-type strain and the $\Delta {relA}$ mutant were suspended in 5 mM glyphosate dissolved in buffered or non-buffered 0.9% NaCl and assayed for cell viability. Buffering was necessary due to the low pH of the glyphosate solution. Figure 2 shows that exposure of wild-type bacteria to 5 mM glyphosate diluted in 0.9% NaCl resulted in the death of 99.9% of cells after 30 minutes. The $\Delta {relA}$ mutant was slightly less sensitive than the wild-type strain, as it took 60 minutes to kill 99.9% of the cells. In contrast, when glyphosate was diluted in Tris buffer (pH 7), both wild-type and $\Delta {relA}$ cells remained viable throughout the entire experiment (6 hours). These results indicate that bacterial death in saline containing 5 mM glyphosate was due to the acidic pH of the solution, which is around 2.0. We can thus conclude that glyphosate is bacteriostatic in both *relA*^+^ and $\Delta {relA}$ bacteria provided that the bacteria are suspended in a medium under physiological pH.

## Discussion

The possible harmful effects of glyphosate on the environment, human and animal health, and on the diversity and physiology of microorganisms are still subject of intense debate. A search in the Google Scholar database revealed almost 20,000 scientific documents with the keywords “glyphosate” and “toxicity” published in the last 10 years. In mammals, several adverse effects, like cancer and/or neurological disorders have been attributed to glyphosate exposure [53]. Glyphosate has also been shown to affect the microbiota in a dose and exposure time-dependent manner [32–35]. It should be pointed out, however, that most of the studies showing adverse effect of glyphosate used exposures levels considerably higher than would be expected to be found in the soil or water bodies in or near agricultural fields or in food consumed by people or animals. In addition, it has been claimed that glyphosate influences the emergence and/or proliferation of bacterial resistance to some antibiotics [36–38]. Yet, the regulatory mechanisms behind glyphosate involvement in bacterial resistance have not yet been fully elucidated. The initial aim of the present study was to test the hypothesis that glyphosate-associated increase in antibiotic resistance is related to the accumulation of (p)ppGpp. However, we determined that in *E. coli* glyphosate does not actually increase the level of antibiotic resistance (Fig. 2). Instead, we hypothesized that glyphosate might affect the tolerance/persistence to antibiotics via (p)ppGpp accumulation.

We confirmed that at a concentration of 5 mM glyphosate exerts a bacteriostatic effect on *E.coli*, both in the wild-type strain and in the $\Delta {relA}$ mutant (Fig. 2). This result facilitated the design of ideal conditions to test the main hypothesis and also showed that an unbuffered glyphosate solution might have a bactericidal effect due to the to the low pH of the formulation used in our lab - technical grade glyphosate with a pH of 2. However, it should be noted that commercial formulations of glyphosate have a pH of 5-6, due to the addition of surfactants and other ingredients.

The time-kill curves and MDK assessments showed that glyphosate increases *E. coli* tolerance and/or persistence towards ampicillin, ciprofloxacin, and kanamycin and that this effect is only partially dependent on the presence of *relA*. More specifically, in both wild-type and $\Delta {relA}$ strains glyphosate increased ciprofloxacin tolerance and persistence, and tolerance toward kanamycin and ampicillin, while in the $\Delta {relA}$ mutant glyphosate also contributed to the formation of persisters in ampicillin-treated cultures. However, it should be noted that in the relaxed strain the protective effect of glyphosate against ciprofloxacin and kanamycin was only slightly diminished and not at all in the case of ampicillin. These results suggest that even though glyphosate induces (p)ppGpp accumulation via RelA and not via SpoT [40], active induction of (p)ppGpp synthesis is not a pre-requisite to tolerance. Whether a basal level of (p)ppGpp provided by the *spoT* gene contributes to the glyphosate-dependent increase in tolerance towards antibiotics remains an open question. This is because testing the effect of glyphosate on the (p)ppGpp^0^ strain ($\Delta {relA}\Delta {spoT}$) is methodologically challenging. Firstly, the MIC of glyphosate in this strain is 40 mM (not shown), close to the maximal solubility of glyphosate in water (about 80 mM). In addition, the (p)ppGpp^0^ strain is poly-auxotrophic having many amino acid requirements and has to be grown in the presence of all 20 amino acids at a concentration of 40 $\upmu$g/ml and a surplus of 400 $\upmu$g/ml serine [54]. It should be noted that addition of aromatic amino acids abolishes the stringent response induced by glyphosate treatment [40]. Lastly, the effect of glyphosate on cell growth and physiology is entirely dependent on *relA* [40].

Unlike *Kurenbach et al. *[36] that reported that glyphosate increases the MIC of ciprofloxacin and kanamycin, we show that glyphosate increases the tolerance to ampicillin, ciprofloxacin and kanamycin, but does not affect the MIC of these antibiotics, i.e., the level of resistance remains unaltered in the presence of glyphosate. However, glyphosate affected the tolerance and/or persistence to the three antibiotics. Here, we relied on the definitions given in the Consensus Statement paper “Definitions and guidelines for research on antibiotic persistence” [11] regarding the concepts of resistance, tolerance, and persistence, and therefore adopted the MDK as the metric to measure tolerance.

It has been demonstrated that (p)ppGpp accumulation induced by amino acid, nitrogen, or glucose starvation plays a fundamental role in the formation of tolerance to certain antibiotics [55] and that this effect can be *relA*-dependent or not [24, 25, 56]. For example, *Kudrin et al.* [56] showed that exposure to mupirocin (an inhibitor of isoleucyl-tRNA synthetase) induces ampicillin tolerance in a *relA*-dependent fashion. However, when mupirocin was combined with trimethoprim the increase in tolerance occurred both in the wild-type and in the relaxed strain. Additionally, the effect of mupirocin on tolerance to norfloxacin was *relA*-independent, and for that reason, they concluded that the effect of (p)ppGpp is drug-specific. The formation of persisters toward ampicillin and ofloxacin was shown to be dependent on (p)ppGpp [24]. Interestingly, deletion of *relA* eliminated persistence towards ampicillin, but ofloxacin persistence was only eliminated when *spoT* was also removed. These authors concluded that persistence to ampicillin requires higher levels of (p)ppGpp than persistence to ofloxacin, and that, in general, tolerance to different antibiotics requires different levels of (p)ppGpp. Similarly, the results presented here show that the increase in tolerance triggered by glyphosate was only partially dependent on *relA*.

The bacteriostatic effect of glyphosate was observed in both wild-type and $\Delta {relA}$ strains [57], which suggests that glyphosate-induced growth inhibition is mostly caused by aromatic amino acid starvation, which is sufficient to halt bacterial growth, even in the absence of (p)ppGpp accumulation. Moreover, it has been argued that there is no specific molecular mechanism involved in persister formation and that any environmental stress that reduces bacterial growth would contribute to the formation of persistence [58]. The relationship between dormant cells and persistence is well known and bacteria that enter into a state of dormancy protect themselves and ensure their survival during upcoming adverse environmental conditions through growth arrest or decreased metabolism [59]. Thus, it is not surprising that a bacterial stressor like glyphosate that conduce bacteria to a dormant state and consequently boosts tolerance or persistence to antibiotics.

The concentration of glyphosate used in this work was the minimal inhibitory concentration in minimal medium – 5 mM or 0.84 mg/ml. While the concentration of glyphosate used in gardening (6 mM) is similar to the concentration used in our experiments, the working concentration of glyphosate spray used in crop fields is around 42 mM. However, the local residue concentrations of glyphosate in soil and plants are considerably lower since glyphosate has a half-life of 1-2 weeks due to microbial degradation [60]. For instance, the maximal concentrations of residual glyphosate found in plant matter were 5 mg/Kg in soybean fields [61] and 0.38 mg/Kg in maize [62], while the maximal concentration of glyphosate in the soil was 2 mg/Kg [63]. These levels are considerably below glyphosate minimal inhibitory concentration and should not cause a significant change in microbial tolerance/persistence. Indeed, concentrations below 1 mM (0.17 mg/L) do not affect *E. coli* growth rate [57] and are unlikely to drive the bacteria into a state of dormancy with the consequent emergence of tolerance. Nevertheless, whether the concentration of glyphosate in natural environments is high enough to increase tolerance or not, our results clearly showed that glyphosate does not increase the MIC of three different antibiotics, but it does play a role on bacterial tolerance or persistence. In principle, any environmental stress that slows down growth rate may potentially lead to tolerance, it is thus not surprising that glyphosate, which causes amino acid starvation, increases bacterial tolerance. In this respect, the use of glyphosate does not generate any more risk to antibiotic failure than any other environmental stress that bacteria encounter during their lifetime.

## Supplementary Information

**Additional file 1.**

## Data Availability

The datasets generated during and/or analysed during the current study are available from the corresponding author on reasonable request.
