# Supplementary material for: Glyphosate affects persistence and tolerance but not antibiotic resistance
Source: BMC Microbiol. 2023 Mar 7;23:61. doi: 10.1186/s12866-023-02804-1 (PMC9990207; doi:10.1186/s12866-023-02804-1)
Supplement: Supplementary file 1 — Additional file 1. [file 12866_2023_2804_MOESM1_ESM.pdf]

Supplement

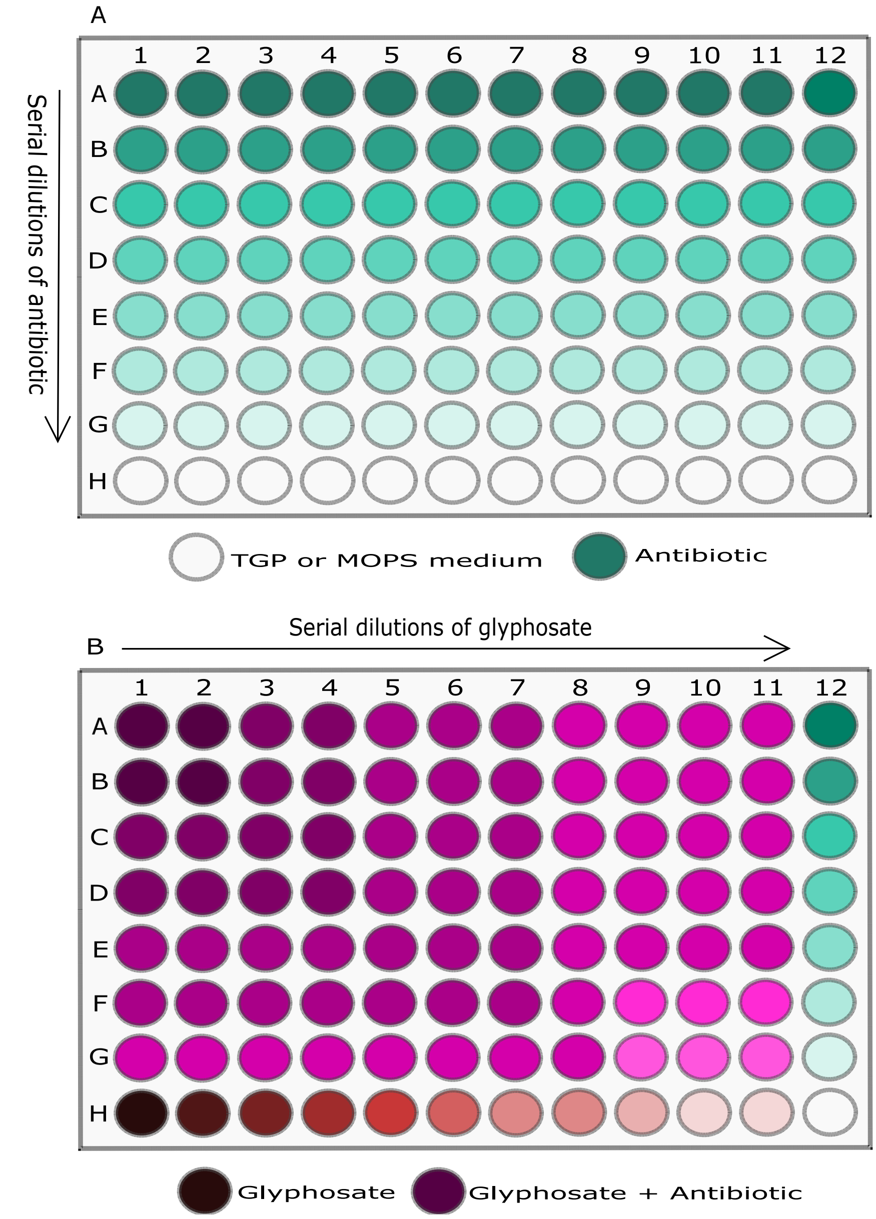

Fig S1: **Chequerboard of antibiotic + glyphosate.** The dilutions of glyphosate and antibiotics were performed on the same plate, but are shown here in separate plates for the sake of clarity. Figure A shows serial dilutions of an antibiotic from row A to G as indicated by the arrow. Row H is empty. Figure B shows serial dilutions of glyphosate from columns 1 to 11. Glyphosate dilutions are made on the 96-well plate in which antibiotics dilutions have been previously added. The result is a microplate containing combinations of different concentrations of glyphosate and antibiotics. Row H contains only glyphosate and column 12 contains only antibiotic.
